# Functional Characterization of Aquaporin-4 Specific T Cells: Towards a Model for Neuromyelitis Optica

**DOI:** 10.1371/journal.pone.0016083

**Published:** 2011-01-14

**Authors:** Sudhakar Reddy Kalluri, Veit Rothhammer, Ori Staszewski, Rajneesh Srivastava, Franziska Petermann, Marco Prinz, Bernhard Hemmer, Thomas Korn

**Affiliations:** 1 Department of Neurology, Klinikum rechts der Isar, Technische Universität München, München, Germany; 2 Department of Neuropathology, Institute of Pathology, Universitätsklinikum Freiburg, Freiburg, Germany; Julius-Maximilians-Universität Würzburg, Germany

## Abstract

**Background:**

Antibodies to the water channel protein aquaporin-4 (AQP4), which is expressed in astrocytic endfeet at the blood brain barrier, have been identified in the serum of Neuromyelitis optica (NMO) patients and are believed to induce damage to astrocytes. However, AQP4 specific T helper cell responses that are required for the generation of anti-AQP4 antibodies and most likely also for the formation of intraparenchymal CNS lesions have not been characterized.

**Methodology/Principal Findings:**

Using overlapping 15-meric peptides of AQP4, we identified the immunogenic T cell epitopes of AQP4 that are restricted to murine major histocompatibility complex (MHC) I-A^b^. The N-terminal region of AQP4 was highly immunogenic. More precisely, the intracellular epitope AQP4_22–36_ was detected as major immunogenic determinant. AQP4_82–108_ (located in the second transmembrane domain), AQP4_139–153_ (located in the second extracellular loop), AQP4_211–225_ (located in the fifth transmembrane domain), AQP4_235–249_ (located in the sixth transmembrane domain), as well as AQP4_289–306_ in the intracellular C-terminal region were also immunogenic epitopes. AQP4_22–36_ and AQP4_289–303_ specific T cells were present in the natural T cell repertoire of wild type C57BL/6 mice and T cell lines were raised. However, active immunization with these AQP4 peptides did not induce signs of spinal cord disease. Rather, sensitization with AQP4 peptides resulted in production of IFN-γ, but also IL-5 and IL-10 by antigen-specific T cells. Consistent with this cytokine profile, the AQP4 specific antibody response upon immunization with full length AQP4 included IgG1 and IgG2, which are associated with a mixed Th2/Th1 T cell response.

**Conclusions and Significance:**

AQP4 is able to induce an autoreactive T cell response. The identification of I-A^b^ restricted AQP4 specific T cell epitopes will allow us to investigate how AQP4 specific autoimmune reactions are regulated and to establish faithful mouse models of NMO that include both cellular and humoral responses against AQP4.

## Introduction

Neuromyelitis optica (Devic syndrome) is a severe inflammatory demyelinating syndrome affecting optic nerves and spinal cord [1]. The detection of NMO-IgG antibodies in the sera of patients with clinically defined NMO but not in patients with multiple sclerosis (MS), gave rise to the concept that NMO might be a distinct disease entity [2]. NMO-IgG reacts against aquaporin-4 (AQP4), a water channel protein that is highly expressed in astrocytic endfeet of the glia limitans [3]. AQP4 is expressed in the CNS, skeletal muscle, lung, kidney, stomach, and exocrine glands (for review see [4]). In the CNS, AQP4 is the main water channel and besides its abundance in astrocytes at the blood/parenchyma barrier, is also expressed in ependymal cells at the CSF/parenchyma barrier. AQP4 KO mice are protected from conditions associated with cytotoxic edema like experimental stroke, but do worse in response to vasogenic edema like in brain tumor models [5], [6]. These data suggest that AQP4 has a role in removing excess water from the CNS interstitial space either by uptake into glial cells or transepithelial transport.

AQP4 has two translational isoforms: a long isoform (M1) in which translation is initiated at Met-1 and a short isoform (M23) in which translation is initiated at Met-23 [7]. M1 and M23 possess 6 putative transmembrane domains with intracellular N- and C-terminal regions and form either homo or heterotetramers [8]. However, only M23 is arranged in large orthogonal arrays of particles (OAP) in the plasma membrane of AQP4 expressing cells [9]. In an mRNA expression study, it has been suggested that M1 and M23 might be differentially expressed in various parts of the CNS with M1 prevailing in the optic nerve and spinal cord and M23 in the brain and cerebellum [10]. It is not known whether M1 and M23 are also differentially targeted by NMO-IgG *in vivo*, which could explain in part the lesion distribution in the spinal cord and optic nerves of NMO patients.

Since the majority (60 to 70%) of patients that are diagnosed with NMO according to clinical criteria are positive for anti-AQP4 antibodies of the IgG1 isotype [2], [11], [12], the presence of NMO-IgG has been incorporated in the diagnostic criteria for NMO [13]. The diagnositic sensitivity and specificity of serum NMO-IgG have been reported to be in the range of 80 to 90% depending on the assay system [2], [11], [14]. Several recent studies were designed to test whether NMO-IgG, beyond its invaluable role as biomarker, was also pathogenetically relevant in NMO. The rationale was that NMO-IgG recognizes an extracellular epitope of AQP4, binds to complement, and leads to the internalization of AQP4 *in vitro* [15]. In addition, plasmapheresis is a beneficial treatment strategy in NMO patients [16]. Indeed, anti-AQP4 IgG1 has now been shown to cause damage to astrocytes *in vivo* as well. First, anti-AQP4-IgG1 and complement deposition can be found in CNS lesions of NMO patients [17], [18]. Second, binding of anti-AQP4 IgG is associated with loss of AQP4 expression and damage to astrocytes [18], [19], [20]. Third, systemic adoptive transfer of AQP4 specific IgG antibodies engineered from intrathecal clones of NMO patients or NMO-IgG serum fractions from patients but not AQP4-preabsorbed serum IgG were able to induce additional perivascular astrocyte loss in experimental rats that had been pretreated with activated myelin specific CD4^+^ T cells to induce disrupture of the blood brain barrier that by itself was subclinical or only mildly symptomatic [21], [22], [23]. Together, these results suggest that NMO-IgG might be involved in the pathogenic process of NMO.

However, the lesions that could be induced in experimental animals by transfer of NMO-IgG lacked the longitudinally extensive properties and parenchymal involvement including myelinolysis that are observed in NMO patients [17] unless NMO-IgG and complement were co-injected directly into the brain in a traumatic approach [23]. Furthermore, intravenous or intraperitoneal transfer of immunoglobulin fractions from NMO patients did not induce astrocytic damage in laboratory animals whose blood brain barrier was leaky in the absence of inflammatory stimuli [22] suggesting that lesion development in NMO may not exclusively rely on effector functions of NMO-IgG. In addition, the generation of anti-AQP4 antibodies of the IgG1 isotype in the peripheral immune compartment inevitably requires class-switch recombination in antigen specific B cells and thus cognate T cell help [24], [25]. Therefore, we hypothesized that there must be an anti-AQP4 specific T cell response in NMO. In the present study, we tested the immunogenicity of AQP4 in C57BL/6 mice and identified the major I-A^b^ restricted immunogenic determinants of AQP4. These data might build the foundation to develop animal models for NMO that include both antigen specific T cell and B cell mediated immunopathology.

## Results

### Identification of I-A^b^ restricted epitopes of AQP4

In humans, AQP4 specific NMO immunoglobulins belong to the IgG1 isotype. Less than 10% of NMO patients have serum IgM antibodies to AQP4 in addition to NMO-IgG1. Thus, class switch recombination must have taken place. A cognate T cell response to AQP4 resulting in subsequent B cell help is required in order to perform the isotype switch from IgM to IgG1. We wanted to characterize the T cell response against AQP4 protein. Thus, we generated lysates from LN18 cells engineered to overexpress full length human AQP4 by lentiviral transduction and purified AQP4 [26]. AQP4 is a highly conserved protein and the protein sequence identity between human and mouse AQP4 M1 and M23 translational isoforms is 92.9% and 94.7%, respectively.

**Figure 1:**
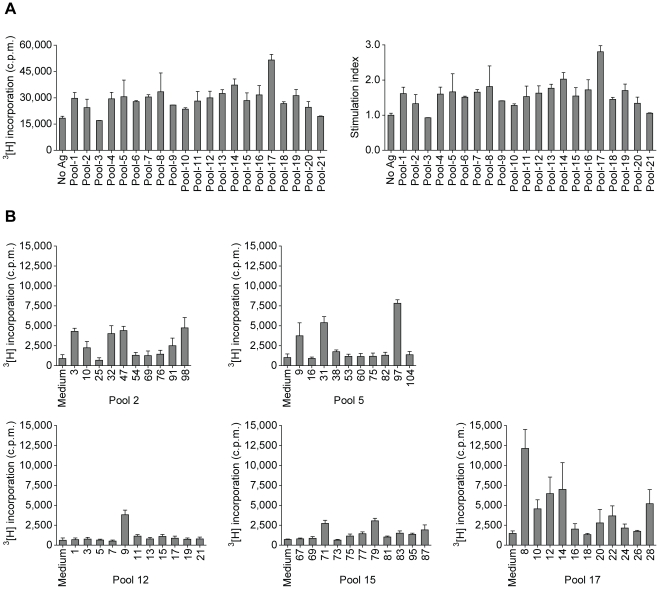
Screening of T cell responses with pools of AQP4 peptides. Mice were immunized with full length AQP4 protein/CFA. Draining lymph node cells and splenocytes were restimulated *in vitro* with pools of overlapping AQP4 peptides (Tables 1 and 2). (A) Proliferative responses of AQP4-sensitized lymphocytes to 21 pools of overlapping AQP4 peptides as measured by ^3^[H] thymidine incorporation (c.p.m.). Mean of triplicate cultures + SD or stimulation indices as calculated by deviding the c.p.m. values of each peptide pool by the background c.p.m. This experiment was performed twice with similar results. (B) In a second round of restimulation, T cells out of each pool were stimulated with the individual peptides of the parental pool in the presence of irradiated syngeneic splenocytes as APCs. Proliferative responses of T cell pools that showed antigen specific responses to at least one individual splenocytes with a stimulation index of at least 3.0 are depicted. Mean of ^3^[H] thymidine incorporation (c.p.m.) of triplicate cultures + SD are shown.

**Figure 2:**
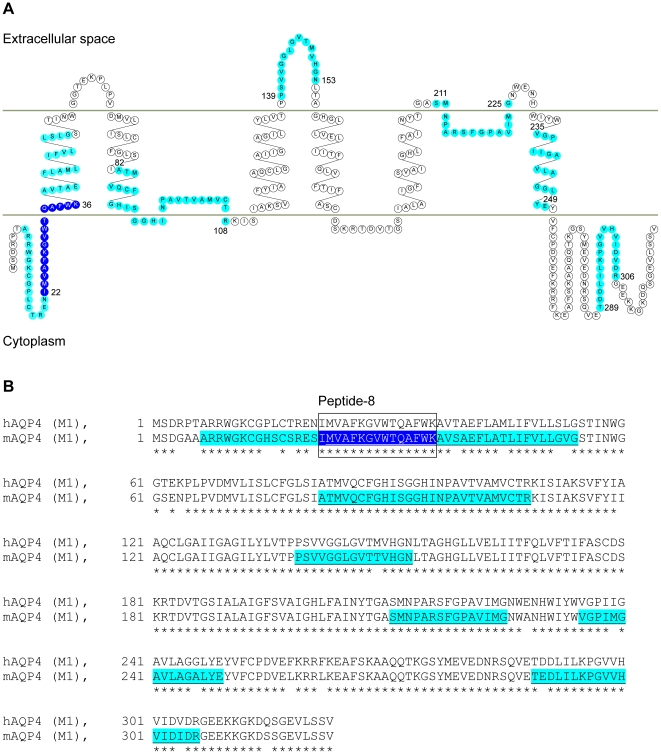
Immunodominant I-A^b^ restricted epitopes of AQP4 and sequence homology of human vs mouse AQP4. (A) Topological model of AQP4 (M1 translational isoform) according to Crane and coworkers [8]. The core immunogenic (I-A^b^ restricted) determinant of AQP4 in the N-terminus is highlighted in dark blue. The remainder of the immunogenic T cell epitopes is highlighted in cyan. The numbers indicate the amino acid residue position in the AQP4 protein sequence. (B) Amino acid sequence alignment of human and mouse AQP4 M1 isoforms. I-A^b^ restricted T cell determinants are highlighted (cyan). The dominant immunogenic epitope is represented by peptide 8 as indicated by a box (peptide sequence in dark blue). T cell epitopes common to M1 and M23 isoforms of AQP4 are underlined. Asterisks indicate sequence identity at the corresponding sequence position.

Full length AQP4 emulsified in CFA was used to immunize wild type C57BL/6 mice (I-A^b^). Draining lymph node cells and splenocytes were isolated and restimulated *in vitro* with pools of overlapping 15-meric peptides spanning the entire M1 AQP4 protein sequence (Tables 1 and 2). While the strongest proliferative response was detected upon restimulation with peptide pool 17 (Fig. 1A), the stimulation indices were only in the range of 1.1 to 3.0 (Fig. 1A). Thus, we decided to restimulate each pool with its individual peptides in a second round. Irradiated syngeneic (H-2K^b^, H-2D^b^, I-A^b^) splenocytes were used as APCs. Any 15-meric peptide that yielded proliferative responses of at least 3 fold above background was considered as potentially immunogenic in the context of I-A^b^ (Fig. 1B). Robust proliferative T cell responses were obtained upon restimulation with epitopes derived from AQP4_8–54_ (peptides 3, 8, 9, 10, 12, 14), AQP4_82–108_ (peptides 28, 31, 32), AQP4_139–153_ (peptide 47), AQP4_211–225_ (peptide 71), AQP4_235–249_ (peptide 79), and AQP4_289–306_ (peptides 97, 98) (Fig. 1B). N-terminal peptide epitopes of AQP4 induced the strongest recall responses (Fig. 1B) suggesting that the N-terminal region of AQP4 comprises the most robust immunogenic I-A^b^ restricted T cell epitopes (Fig. 2A). The core immunogenic N-terminal determinant of AQP4 (AQP4_22–36_ = peptide 8) is common to both the M1 and M23 translational isoforms of AQP4, except for the isoleucine at positon 22, which is absent in the M23 isoform of AQP4. The amino acid sequence of AQP4_22–36_ is identical between human and mouse while there is only minimal amino acid variance between human and mouse in the additional I-A^b^ restricted AQP4 epitopes (Fig. 2B). Thus, AQP4 is immunogenic in the I-A^b^ context and wild type C57BL/6 mice harbor autoreactive AQP4 specific T cells in their natural repertoire.

**Table 1:** Overlapping AQP4 peptides.

No	AA	Sequence	No	AA	Sequence	No	AA	Sequence
1	1–15	MSDRPTARRWGKCGP	36	106–120	CTRKISIAKSVFYIA	71	211–225	SMNPARSFGPAVIMG
2	4–18	RPTARRWGKCGPLCT	37	109–123	KISIAKSVFYIAAQC	72	214–228	PARSFGPAVIMGNWE
3	7–21	ARRWGKCGPLCTREN	38	112–126	IAKSVFYIAAQCLGA	73	217–231	SFGPAVIMGNWENHW
4	10–24	WGKCGPLCTRENIMV	39	115–129	SVFYIAAQCLGAIIG	74	220–234	PAVIMGNWENHWIYW
5	13–27	CGPLCTRENIMVAFK	40	118–132	YIAAQCLGAIIGAGI	75	223–237	IMGNWENHWIYWVGP
6	16–30	LCTRENIMVAFKGVW	41	121–135	AQCLGAIIGAGILYL	76	226–240	NWENHWIYWVGPIIG
7	19–33	RENIMVAFKGVWTQA	42	124–138	LGAIIGAGILYLVTP	77	229–243	NHWIYWVGPIIGAVL
8	22–36	IMVAFKGVWTQAFWK	43	127–141	IIGAGILYLVTPPSV	78	232–246	IYWVGPIIGAVLAGG
9	25–39	AFKGVWTQAFWKAVT	44	130–144	AGILYLVTPPSVVGG	79	235–249	VGPIIGAVLAGGLYE
10	28–42	GVWTQAFWKAVTAEF	45	133–147	LYLVTPPSVVGGLGV	80	238–252	IIGAVLAGGLYEYVF
11	31–45	TQAFWKAVTAEFLAM	46	136–150	VTPPSVVGGLGVTMV	81	241–255	AVLAGGLYEYVFCPD
12	34–48	FWKAVTAEFLAMLIF	47	139–153	PSVVGGLGVTMVHGN	82	244–258	AGGLYEYVFCPDVEF
13	37–51	AVTAEFLAMLIFVLL	48	142–156	VGGLGVTMVHGNLTA	83	247–261	LYEYVFCPDVEFKRR
14	40–54	AEFLAMLIFVLLSLG	49	145–159	LGVTMVHGNLTAGHG	84	250–264	YVFCPDVEFKRRFKE
15	43–57	LAMLIFVLLSLGSTI	50	148–162	TMVHGNLTAGHGLLV	85	253–267	CPDVEFKRRFKEAFS
16	46–60	LIFVLLSLGSTINWG	51	151–165	HGNLTAGHGLLVELI	86	256–270	VEFKRRFKEAFSKAA
17	49–63	VLLSLGSTINWGGTE	52	154–168	LTAGHGLLVELIITF	87	259–273	KRRFKEAFSKAAQQT
18	52–66	SLGSTINWGGTEKPL	53	157–171	GHGLLVELIITFQLV	88	262–276	FKEAFSKAAQQTKGS
19	55–69	STINWGGTEKPLPVD	54	160–174	LLVELIITFQLVFTI	89	265–279	AFSKAAQQTKGSYME
20	58–72	NWGGTEKPLPVDMVL	55	163–177	ELIITFQLVFTIFAS	90	268–282	KAAQQTKGSYMEVED
21	61–75	GTEKPLPVDMVLISL	56	166–180	ITFQLVFTIFASCDS	91	271–285	QQTKGSYMEVEDNRS
22	64–78	KPLPVDMVLISLCFG	57	169–183	QLVFTIFASCDSKRT	92	274–288	KGSYMEVEDNRSQVE
23	67–81	PVDMVLISLCFGLSI	58	172–186	FTIFASCDSKRTDVT	93	277–291	YMEVEDNRSQVETDD
24	70–84	MVLISLCFGLSIATM	59	175–189	FASCDSKRTDVTGSI	94	280–294	VEDNRSQVETDDLIL
25	73–87	ISLCFGLSIATMVQC	60	178–192	CDSKRTDVTGSIALA	95	283–297	NRSQVETDDLILKPG
26	76–90	CFGLSIATMVQCFGH	61	181–195	KRTDVTGSIALAIGF	96	286–300	QVETDDLILKPGVVH
27	79–93	LSIATMVQCFGHISG	62	184–198	DVTGSIALAIGFSVA	97	289–303	TDDLILKPGVVHVID
28	82–96	ATMVQCFGHISGGHI	63	187–201	GSIALAIGFSVAIGH	98	292–306	LILKPGVVHVIDVDR
29	85–99	VQCFGHISGGHINPA	64	190–204	ALAIGFSVAIGHLFA	99	295–309	KPGVVHVIDVDRGEE
30	88–102	FGHISGGHINPAVTV	65	193–207	IGFSVAIGHLFAINY	100	298–312	VVHVIDVDRGEEKKG
31	91–105	ISGGHINPAVTVAMV	66	196–210	SVAIGHLFAINYTGA	101	301–315	VIDVDRGEEKKGKDQ
32	94–108	GHINPAVTVAMVCTR	67	199–213	IGHLFAINYTGASMN	102	304–318	VDRGEEKKGKDQSGE
33	97–111	NPAVTVAMVCTRKIS	68	202–216	LFAINYTGASMNPAR	103	307–321	GEEKKGKDQSGEVLS
34	100–114	VTVAMVCTRKISIAK	69	205–219	INYTGASMNPARSFG	104	309–323	EKKGKDQSGEVLSSV
35	103–117	AMVCTRKISIAKSVF	70	208–222	TGASMNPARSFGPAV			

**Table 2:** Peptide pools.

Pool	1	2	3	4	5	6	7	8	9	10	11
12	1	3	5	7	9	11	13	15	17	19	21
13	23	25	27	29	31	33	35	37	39	41	43
14	45	47	49	51	53	55	57	59	61	63	65
15	67	69	71	73	75	77	79	81	83	85	87
16	89	91	93	95	97	99	101	103	2	4	6
17	8	10	12	14	16	18	20	22	24	26	28
18	30	32	34	36	38	40	42	44	46	48	50
19	52	54	56	58	60	62	64	66	68	70	72
20	74	76	78	80	82	84	86	88	90	92	94
21	96	98	100	102	104						

### Characterization of AQP4_22–36_ specific T cell lines

**Figure 3:**
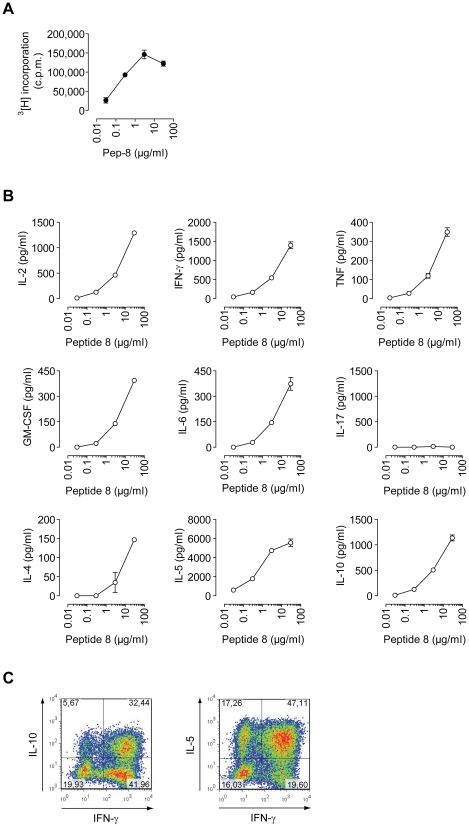
Functional characterization of AQP4_22–36_ peptide specific TCL8.2. Representative T cell line (TCL) reactive to AQP4_22–36_. (A) TCL8.2 cells were restimulated with irradiated syngeneic splenocytes in the presence of increasing concentrations of AQP4_22–36_. The proliferative response was determined by ^3^[H] thymidine incorporation. Mean c.p.m. of triplicate cultures + SD. (B) Cytokine production of TCL8.2 cells in response to increasing concentrations of AQP4_22–36_ as measured by cytometric bead array in the cell culture supernatant collected at 48 h after initiation of restimulation. Mean cytokine concentrations + SD of triplicate cultures. (C) Intracellular cytokine staining of TCL8.2 after 6 restimulation cycles.

AQP4_22–36_ proved to be immunodominant and the proliferative response of short term T cell cultures from lymph node cells of AQP4_22–36_ immunized mice were very robust (Fig. 3A). In order to qualitatively characterize AQP4_22–36_ specific T cell responses, we tested the cytokine profile of lymph node cells from AQP4_22–36_/CFA-sensitized mice upon restimulation with AQP4_22–36_ *in vitro*. Although we used CFA as adjuvant, the first rounds of peptide specific restimulation of lymph node cells under neutral conditions yielded mixed Th1/Th2 cytokine responses (Fig. 3B). We observed production of IL-2, IFN-γ, TNF, GM-CSF, IL-6, but also IL-4 and large amounts of IL-5 and IL-10 while IL-17 was not detectable (Fig. 3B). About one third of the responding T cells were IFN-γ^+^IL-10^+^ and close to one half were IFN-γ^+^IL-5^+^ (Fig. 3C). Together, these data suggest that in our experimental system, AQP4_22–36_ induced the expansion of T cells with a unique cytokine profile that was characterized by co-expression of large amounts of Th1 and Th2 associated cytokines.

### Immunization with AQP4 peptides does not induce clinical disease

**Figure 4:**
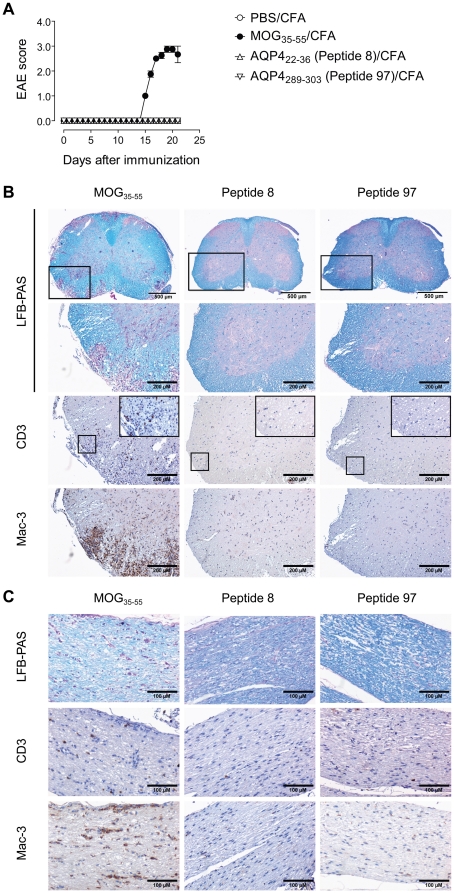
Immunization of C57BL/6 mice with AQP4_22–36_ or AQP4_289–303_ does not induce signs of spinal cord disease or optic neuritis. Groups of wild type C57BL/6 mice (n = 4 per group) were immunized with PBS/CFA, MOG_35–55_/CFA, AQP4_22–36_ (peptide 8)/CFA, or AQP4_289–303_ (peptide 97)/CFA. (A) EAE scores. Note that AQP4 peptide immunized mice did not develop signs of disease while MOG_35–55_/CFA immunized animals showed classical paraparesis. (B, C) Mice that had been immunized with MOG_35–55_/CFA, AQP4_22–36_ (peptide 8)/CFA, or AQP4_289–303_ (peptide 97)/CFA were analysed by histology. Representative sections of spinal cords (B) and optic nerves (C) are shown. Paraffin sections were stained with luxol fast blue (LFB)-PAS to illustrate demyelination or with antibodies to CD3 or Mac-3 to visualize T cell or macrophage infiltrates, respectively. Spinal cord areas that are shown at higher magnification are marked by frames. Scale bars are 500 µm and 200 µm for the spinal cord sections and 100 µm for the optic nerve sections as indicated.

AQP4 specific T cell responses were characterized by a mixed Th1/Th2 phenotype. We predicted that the exaggerated IL-10 production by AQP4_22–36_ specific T cells would prevent T cell driven immunopathology in AQP4 immunized animals. In order to test whether active immunization with AQP4 in CFA was able to induce clinical signs of central nervous damage, we sensitized C57BL/6 mice with full length AQP4/CFA, AQP4_22–36_ (peptide 8)/CFA, or AQP4_289–303_ (peptide 97)/CFA plus administration of pertussis toxin and followed the animals for 4 weeks. While all mice in a MOG_35–55_/CFA immunized control group developed severe EAE, none of the AQP4 immunization protocols resulted in the induction of a clinically manifest spinal cord disease or signs of visual impairment (Fig. 4A). Neither did we observe weight loss or signs of kidney disease. The animals remained apparently healthy and after four weeks were subjected to histological work-up. Here, neither cellular infiltrates nor myelin loss were identified in the spinal cords or optic nerves of AQP4 peptide immunized animals (Fig. 4B, C). Thus, despite a strong antigen specific T cell response against AQP4, the animals remained protected from immunopathology in AQP4 expressing tissues.

### T helper cell responses to AQP4 shape a specific AQP4 specific IgG response pattern

**Figure 5:**
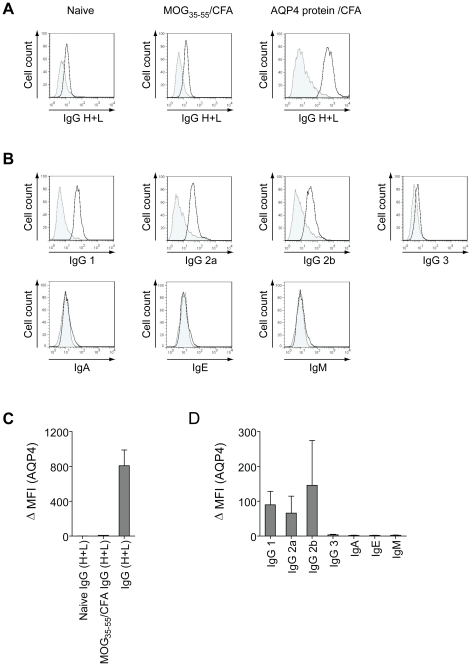
Antibody response to AQP4. Wild type C57BL/6 mice were immunized with full length AQP4/CFA as described in Materials and Methods. Unimmunized mice or mice immunized with MOG_35–55_/CFA were used as controls. Sera of mice from each group were collected and tested for AQP4 specific antibodies in a cell based flow cytometry analysis. Subclass specification was performed by using fluorochrome labeled anti-mouse Ig antibodies specific for IgM, IgA, IgE, IgG1, IgG2a, IgG2b, and IgG3 (FITC labeled) or total IgG H+L (AlexaFluor488 labeled). (A) Representative histogram plots illustrating the MFIs for AQP4 specific IgG H+L in the various test groups. (B) Representative histogram plots of AQP4 specific Ig classes and IgG subclasses in AQP4 immunized animals. (C) ΔMFIs + SEM (n = 4) for AQP4 specific IgG H+L in naive control mice, MOG_35–55_ immunized mice, or full length AQP4 immunized mice. (D) ΔMFIs + SEM (n = 4) for individual anti-AQP4 Ig classes and IgG subclasses in full length AQP4 immunized mice.

We wished to validate whether the cytokine pattern of AQP4 specific T cell responses was relevant to the adaptive immune response to AQP4 *in vivo*. The bias of antigen specific T helper cell responses can be determined *in vivo* by measuring the IgG subtypes that are produced by antigen specific B cells in a T helper cell dependent manner. It is known that in mice, Th1 biased T helper cell responses favor the production of antigen specific IgG2a and IgG3 while Th2 responses prompt B cells to undergo class switch recombination towards IgG1 and IgE [24], [25], [27]. Immunization with AQP4 protein/CFA but not with MOG_35–55_/CFA evoked a robust anti-AQP4 IgG response which was measured in the sera of sensitized mice (Fig. 5A). While AQP4 specific IgA, IgE, and IgM could not be detected, the dominant IgG subclasses were IgG1, IgG2a, and IgG2b but not IgG3 (Fig. 5B, C) indicating that the T helper cell response to AQP4/CFA in this immunization regimen was a mixed Th1/Th2 response *in vivo* as well.

In summary, these data suggest that the antibody response to AQP4 is T cell-dependent. Both direct analysis of T cell responses to AQP4 and measurement of antigen specific Ig subclass patterns *in vivo* highlight a mixed Th1/Th2 response upon immunization with AQP4 protein.

## Discussion

In the present study, we provide evidence that an autoreactive T cell response against AQP4 can be induced in C57BL/6 mice and we determined the I-A^b^ restricted T cell epitopes. While AQP4_22–36_ is the dominant T cell epitope, there are other immunogenic regions in the AQP4 sequence. With the exception of the isoleucine at position 22 of AQP4_22–36_, all T cell epitopes are contained in both M1 and M23 translational isoforms of AQP4. The AQP4 specific T cell response likely accounts for the T cell supported class switch recombination of AQP4 specific B cells and the production of anti-AQP4 IgG type antibodies.

Recent attempts to prove the pathogenic relevance of NMO-IgG have focused on rats as recipients of purified IgG fractions from NMO-patients because rat but not mouse complement binds to purified human NMO-IgG [23] and thus, NMO-IgG dependent CNS damage can easier be shown in recipient rats than in mice receiving human serum IgG. Indeed, passive transfer of NMO-IgG or engineered monoclonal antibodies against AQP4 resulted in extinction of AQP4 in astrocytic endfeet and perhaps in astrocyte loss [21], [22]. Myelin specific T cell blasts but not non-inflammatory stimuli were able to promote the pathogenic potential of NMO-IgG presumably by disrupting the blood brain barrier. In contrast, a recent report suggested that upon immunization with CFA alone, i. e. in the absence of a CNS-antigen specific T cell response, astrocyte damage could be induced by transfer of IgG fractions from NMO patients [28]. However, in all these transfer experiments, the CNS lesions induced by NMO-IgG were distinct from lesions found in human Devic's patients and in particular, failed to recapitulate a vast extent of demyelination [17].

We believe that the identification of the AQP4 determinants that evoke I-A^b^ restricted T cell responses is a further step towards the development of an animal model for Devic's disease that would incorporate both T cell and B cell mediated anti-AQP4 responses. The current concept of neuromyelitis optica is based on the idea that this disease might be an astrocyte disease with astrocytic AQP4 as a major molecular target of the adaptive immune response. We proposed that there must be an anti-AQP4 T cell response to account for the induction of class switched antibodies to AQP4. Here, we identified T cell epitopes of AQP4 in the context of I-A^b^. AQP4 specific T cell responses were elicited in wild type mice. Thus, our data suggest that - similar to other autoantigens - thymic deletional tolerance against AQP4 is incomplete. T cell lines raised against AQP4_22–36_, the major immunogenic epitope of AQP4, produced IFN-γ but were consistently also characterized by simultaneous secretion of Th2 associated cytokines. Accordingly, immunization with full length AQP4 protein induced a T cell dependent AQP4 specific antibody response whose Ig class and subclass pattern was reminiscent of a mixed Th1/Th2 reaction. Although T cell tolerance against AQP4 is broken upon immunization with AQP4/CFA, it is likely that AQP4 directed immunopathology is limited due to T cell derived IL-10. We are currently testing this hypothesis in further experiments.

Excellent animal models that include both CNS antigen specific T and B cell responses have recently been developed for MOG as target autoantigen. Transgenic mice that harbor both MOG specific T cells and MOG specific B cells develop a severe opticospinal syndrome and were proposed to mimic certain aspects of Devic's disease [29], [30]. Many aspects of the cooperation between T and B cells, namely the capacity of antigen specific B cells to function as APCs for T cells of the same specificity, were extensively investigated in these animal models. In addition, MOG specific T cell driven immunopathology in the CNS appears to recruit MOG specific B cells from the natural repertoire to develop into antibody producing cells and contribute to lesion development by MOG specific antibodies [31]. These data highlight the pathogenic importance of a CD4^+^ T helper cell response to an autoantigen that is identical or locally linked to the respective B cell autoantigen. However, with the discovery and validation of NMO-IgG in reductionist *in vitro* and *in vivo* models, it has become evident that astrocytic AQP4 may be the relevant target autoantigen in neuromyelitis optica instead of MOG which is expressed by oligodendrocytes [32].

The characterization of the I-A^b^ restricted AQP4 T cell epitopes, will now allow us to investigate the mechanisms that result in the breach of immunological tolerance against AQP4 and also why immunopathology develops in the CNS while other AQP4 expressing tissues are relatively spared. Based on the current results, we may be able to construct animal models in mice that may give us insight in the pathogenic cascade of autoimmune inflammatory astrocyte diseases and perhaps help us to understand how astrocyte targeted T and B cell responses ultimately lead to extensive CNS lesions.

## Materials and Methods

### Aquaporin-4 protein expression and purification

The human M1 variant of AQP4 protein was stably overexpressed in LN18 cells (LN18^AQP4^) by lentiviral transfection as described previously [26]. In order to generate AQP4 protein, cells were grown at large scale and proteins were extracted from LN18^AQP4^ cells by using mammalian protein extraction reagent buffer (Thermo Scientific) according to the manufacturer's recommendations. AQP4 protein was isolated by column-based affinity purification using a polyclonal rabbit anti human-AQP4 antibody (Sigma) coupled to cyanogen bromide-activated sepharose 4B according to the manufacturer's instructions (Amersham pharmaci). Yield and purity of the protein preparation was tested by coomassie staining and western blot of SDS-PAGE gels.

### Peptide antigens

Mouse MOG_35–55_ (MEVGWYRSPFSRVVHLYRNGK) and human AQP4_22–36_ (IMVAFKGVWTQAFWK) as well as human AQP4_289–303_ (TDDLILKPGVVHVID) were synthesized by Auspep Pty Ltd (Parkville, Victoria, Australia) and JPT Peptide Technology (Berlin, Germany), respectively. Overlapping peptides (15-mers) of full length human AQP4 protein (M1 isoform) were synthesized by JPT Peptide Technology according to Table 1.

### Mice and immunization procedures

Wild type C57BL/6 mice (H-2K^b^, H-2D^b^, I-A^b^) at 8 weeks of age were immunized subcutaneously with an emulsion of antigen in complete Freund's adjuvant (CFA) with a final concentration of 2.5 mg/ml mycobacterium extract H37Ra. Individual emulsions contained final antigen concentrations of 1 mg/ml for MOG_35–55_ peptide, AQP4_22–36_ peptide, AQP4_289–303_ peptide, or AQP4 protein, respectively. Each mouse received a total volume of 100 µl of immunization inoculum corresponding to 100 µg of antigen as subcutaneous injections at the base of the tail. In addition, mice were administered 200 ng pertussis toxin in PBS i. p. on days 0 and 2 after immunization. When immunized with full length AQP4 protein, mice received a booster immunization with AQP4 protein/CFA (in the absence of additional pertussis toxin) after 4 weeks. Where indicated, clinical signs of EAE were assessed according to following score: 0, no sings of disease; 1, loss of tone in the tail; 2, hind limb paresis; 3, hind limb paralysis; 4, tetraplegia; 5, moribund. Mice were kept in a pathogen free facility at the Neurological Department of the Technical University Munich. The animal protocol was approved by the animal welfare committee of the Bavarian state authorities (Tierschutzkommission der Regierung von Oberbayern, Munich, Germany, License number: 55.2-1-54-2531-88-08). All experiments were carried out in accordance with the guidelines prescribed by the Bavarian state authorities.

### AQP4 peptide pool screening, antigen specific restimulation and proliferation assays

Draining lymph node cells and splenocytes were isolated from full length AQP4/CFA-immunized mice on day 6 after the booster immunization. Peptide pools were generated according to Table 2 with each peptide at a final concentration of 10 µg/ml. In a 96 well format, suspensions of 400,000 *in vivo* sensitized mixed lymph node and spleen cells were re-stimulated for 72 h *in vitro* with individual peptide pools in DMEM/10% FCS supplemented with 5×10^−5^ M β-mercaptoethanol, 1 mM sodium pyruvate, non-essential amino acids, L-glutamine, and 100 U penicillin/100 µg streptomycin per ml (clone medium). During the last 16 h, cells were pulsed with 1 µCi of ^3^[H]thymidine (PerkinElmer) followed by harvesting on glass fiber filters and analysis of incorporated ^3^[H]thymidine in a β-counter (1450 Microbeta, Trilux, PerkinElmer). In a parallel set-up, peptide pool restimulations were supplementated with recombinant mouse IL-2 at 2 ng/ml (R&D systems). After 14 days, cells from each peptide pool were divided in aliquots and each aliquot was restimulated in clone medium with a single peptide out of the parental pool at a concentration of 10 µg/ml in clone medium in the presence of irradiated (3000 rad) splenic APCs isolated from naive C57BL/6 mice. After 48 h, supernatants were collected for cytokine measurement and proliferation was determined by ^3^[H]thymidine incorporation as described.

T cell lines specific for immunogenic determinants of AQP4 were generated from draining lymph node cells isolated 2 weeks after immunization from mice immunized with AQP4_22–36_/CFA or AQP4_289–303_/CFA. In cyclic recall stimulations, draining lymph node cells were stimulated with AQP4_22–36_ or AQP4_289–303_ in clone medium in the presence of irradiated syngeneic splenic APCs. Restimulation cycles with irradiated splenic APCs were repeated every 10 to 12 days to obtain antigen specific T cell lines. In order to propagate the T cell lines, recombinant mouse IL-2 was added at a concentration of 2 ng/ml 72 h after set-up of antigen specific recall cultures.

### Cytokine profiling of AQP4 peptide specific T cell lines

Supernatants of antigen specific T cell lines were assessed for cytokine concentrations with standard ELISA technique (R&D systems) or cytometric bead array (Bender systems) according to the manufacturers' recommendations. For intracellular cytokine staining, AQP4 peptide specific T cell lines were stimulated in clone medium containing phorbol 12-myristate 13-acetate (PMA, 50 ng/ml, Sigma), ionomycin (1 µg/ml, Sigma), and monensin (GolgiStop 1 µl/ml, BD Biosciences) at 37°C/10% CO_2_ for 4 h. After staining of surface markers, cells were fixed and permeabilized (Cytofix/Cytoperm and Perm/Wash buffer, BD Biosciences) followed by staining with monoclonal antibodies to mouse IL-4, IL-5, IL-10, IL-17, and IFN-γ, (all obtained from BD Biosciences) and fluorocytometric analysis (CYAN, Beckmann/Coulter).

### Determination of anti-AQP4 antibody subtypes in mouse sera of immunized mice

To determine antibody titres and Ig subtypes against AQP4, sera were collected from PBS/CFA-, MOG_35–55_/CFA-, or AQP4 protein/CFA-immunized mice. Serum samples were taken 3 months after initial sensitization of mice. Sera were diluted in 1: 50 in PBS 1% FCS and applied for detection of conformational antibodies to AQP4 in a cell based assay previously described [26]. Briefly, AQP4 transduced LN18 cells (LN18^AQP4^) or control transduced LN18 cells (LN18^CTR^) were exposed to the diluted sera. Ig subclasses were identified by using AlexaFluor488 labelled secondary antibodies to mouse IgG H+L (Invitrogen) or FITC labeled anti-IgM, -IgA, -IgE, -IgG1, -IgG2a, -IgG2b, or -IgG3, (Serotec). Staining of LN18^AQP4^ or control cells was analysed by flow cytometry. Antibody titres were calculated based on delta mean fluorescence intensity (ΔMFI) of LN18^AQP4^ vs LN18^CTR^.

### Histology

Mice were sacrificed using CO_2_. Histology was performed as described recently [33], [34]. Spinal cords including spinal canal and optic nerves were removed and fixed in 4% buffered formalin. Then, spinal cords were dissected and embedded in paraffin before staining with luxol fast blue (LFB) to assess the degree of demyelination, anti-MAC-3 (BD Biosciences) for assessment of macrophages/microglia infiltrates, and anti-CD3 for assessment of T cell infiltrates (Serotec).

### Statistical analysis

Statistical evaluations of cell frequency measurements and proliferation data were performed using the unpaired Student's t-test. For comparison of clinical EAE scores, the Mann-Whitney U rank sum test was used. P values<0.05 were considered significant.
